# Unusual Subcutaneous Presentation of Cavernous Hemangioma in the Lower Eyelid: A complete translation from Farsi

**DOI:** 10.18502/jovr.v15i2.6741

**Published:** 2020-04-06

**Authors:** Abbas Bagheri, Mohaddeseh Feizi, Mehdi Tavakoli

**Affiliations:** ^1^ Ocular Tissue Engineering Research Center, Shahid Beheshti University of Medical Sciences, Tehran, Iran; ^2^ Ophthalmic Research Center, Shahid Beheshti University of Medical Sciences, Tehran, Iran; ^3^ Department of Ophthalmology, Labbafinejad Medical Center, Shahid Beheshti University of Medical Sciences, Tehran, Iran; ^4^ Department of Ophthalmology, The University of Alabama at Birmingham, Callahan Eye Hospital, Birmingham, Al, USA

**Keywords:** Cavernous Hemangioma, Eyelid Mass, Lower Eyelid Mass

## Abstract

**Purpose:**

To report a patient with cavernous hemangioma (CH) presenting as a “subcutaneous” lower eyelid mass.

**Case report:**

A 37-year-old man presented with a painless and palpable mass over the right lower eyelid for two years prior to referral. Computed tomography scan revealed a well-defined, lobulated mass located in the mid and lateral portion of the lower eyelid that extended posteriorly to the anterior orbital space. A transcutaneous excisional biopsy was performed. Histopathologic findings of the tumor confirmed CH. Most CHs are intraconal lesions, making our case an unusual presentation for this condition.

**Conclusion:**

**Purpose:**

**Case report:**

CH may present superficially in the eyelid and anterior orbital area and thus, although this location is not common, it should be kept in mind as a differential diagnosis for any well-defined eyelid tumor.

##  INTRODUCTION

Cavernous hemangioma (CH) is the most common benign orbital tumor in adults. It is most commonly seen in middle-aged women and usually presents as a gradually increasing, painless, axial proptosis with a single well-defined and slow-growing intraconal mass.^[1,2,3]^

CH can also infrequently develop in the extraconal orbital space producing non-axial proptosis. The involvement of adnexal tissues including conjunctiva, lacrimal gland, and even orbital bones has rarely been reported. To the best of our knowledge, there are very few reports of eyelid CH in the literature.^[4,5,6,7,8]^ Herein, we report a patient with eyelid CH presenting as a subcutaneous lower eyelid mass.

**Figure 1:**
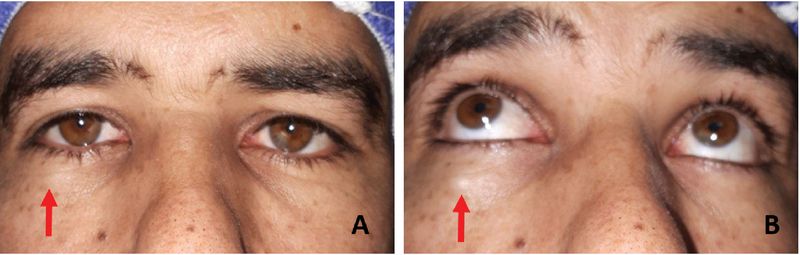
Clinical photography of the patient showing a nodular subcutaneous mass about 1 cm below the right lower lid margin which is more prominent in the upward gaze and skin depigmentation overlying the lesion. (Arrow shows the lesion) (A) Primary position. (B) Upward gaze.

**Figure 2:**
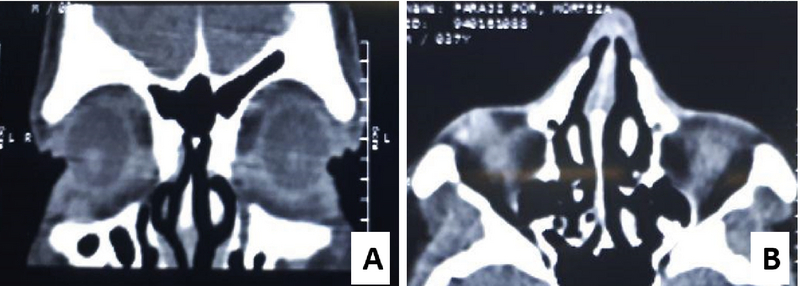
Orbital CT scan shows a well-defined mass in the inferotemporal extraconal space of the right orbit which has a single lobe posteriorly and changes to bilobed anteriorly. (A) Coronal view of the anterior orbital space. (B) Axial view of the inferior orbital space.

**Figure 3:**
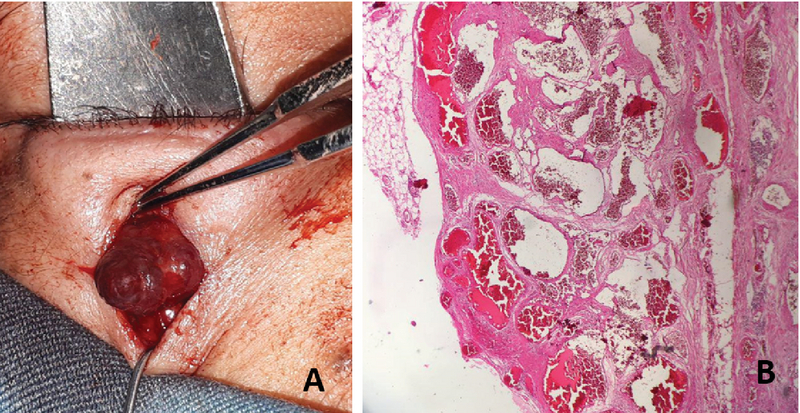
(A) Intraoperative view of the mass shows a dark-red encapsulated mass which is seen bilobed in the anterior view. (B) Microscopic pathology shows dilated thick wall spaces with a single layer of endothelium loaded with blood cells and thrombosis and a well-defined capsule typical of orbital cavernous hemangioma (Hematoxylin and Eosin, magnification × 100).

##  CASE REPORT

A 37-year-old man presented with a painless mass on the right lower eyelid, which gradually grew over two years prior to referral. He reported no fluctuation in the size of the mass with Valsalva maneuver or exercises.

On examination, a nodular subcutaneous lesion was noted slightly lateral to the midline of the right lower lid, and about 1 cm inferior to the lid margin. The lesion became more prominent in upward gaze and a small area of skin depigmentation was observed overlying the lesion [Figures 1(a) and 1(b)].

Palpation of the mass revealed a firm, freely mobile subcutaneous mass. No proptosis was noted. Visual acuity, extraocular muscle movements, and pupillary reflexes were normal. Anterior and posterior segment examinations were also within normal limits.

CT scan of the orbit showed a lobulated soft tissue mass with a well-defined border in the temporal area of the right lower lid, which extended to the extraconal inferior orbital space. The mass had one lobe posteriorly and was bilobed anteriorly [Figures 2(a) and 2(b)]. The patient refused imaging with contrast.

The lesion was removed using a transcutaneous approach over the mass by blunt dissection while being pushed anteriorly by a malleable retractor in the lower lid fornix. A dark violet well-encapsulated mass measuring 1.3 × 1.2 × 1 cm was excised. The mass had two anterior fused lobes which unified into a single lobe posteriorly [Figure 3(a)]. Histopathologic examination showed dilated thick wall spaces with a single layer of endothelium loaded with blood cells and areas of thrombosis. There was also a hypercellular stroma and a well-defined capsule. These histological findings were consistent with CH [Figure 3(b)].

The operation and postoperative course were uneventful. Follow-up was done in 12 months.

##  DISCUSSION

The origin of CH is controversial. Some authors classify CH as low flow venous malformations, while others believe that CH lesions are indeed low flow arteriovenous malformations.^[1,2,3][9]^ Orbital CH is essentially a gradual growing lesion; however, it has been shown that CH has a more rapid growth in extraconal locations and in male subjects.^[10]^ As was seen in our case, CH has a predilection for lateral and inferior orbital spaces, which is attributed to the rich arterial flow in the area.^[11]^

CH is one of the common vascular lesions in the liver and subcutaneous tissues, but in those areas, they typically have no prominent capsule. This is in contrast to the orbital space, in which a well-defined capsule is a characteristic feature.^[9,12]^ In our case, although presenting with a subcutaneous mass, the presence of a well-defined capsule was in favor of the orbit as the origin of the CH. This means that a CH may have some characteristics of subcutaneous CH and other characteristics of its orbital counterpart.

Eyelid presentations have rarely been reported in large studies of orbital CH. A palpable mass was reported in 19.6% of patients in a study on 214 cases of orbital CH.^[13]^ However, orbital CH extending to the subcutaneous areas was seen in 9.3% and 5.1% of the patients in the studies by Aymard et al^[14]^ and Rootman et al,^[1]^ respectively.

In most previously reported cases, eyelid CH lesions were located in the upper lid subcutaneous area ^[4,5,6,7]^, whereas, there was only one case report of lower eyelid CH^[8]^ in which the patient complained of proptosis and not an eyelid mass.

Finally, although CH of the eyelid is unusual, it should be considered in the list of differential diagnosis of subcutaneous eyelid lesions and surgeons should be prepared for a vascular lesion.

##  Financial Support and Sponsorship

None.

##  Conflicts of Interest

There are no conflicts of interest.
